# Fluorophore-Probed Curdlan Polysaccharide Chemosensor: “Turn-On” Oligosaccharide Sensing in Aqueous Media

**DOI:** 10.1021/acsomega.4c01786

**Published:** 2024-05-13

**Authors:** Masahiro Norikuni, Yumiko Hori, Munenori Numata, Michiya Matsusaki, Toshiyuki Kida, Gaku Fukuhara

**Affiliations:** † Department of Applied Chemistry, Osaka University, 2-1 Yamada-oka, Suita 565-0871, Japan; ‡ Department of Chemistry, Tokyo Institute of Technology, 2-12-1 Ookayama, Meguro-ku, Tokyo 152-8551, Japan; § Department of Biomolecular Chemistry, Graduate School of Life and Environmental Sciences, Kyoto Prefectural University, Shimogamo Sakyo-ku, Kyoto 606-8522, Japan

## Abstract

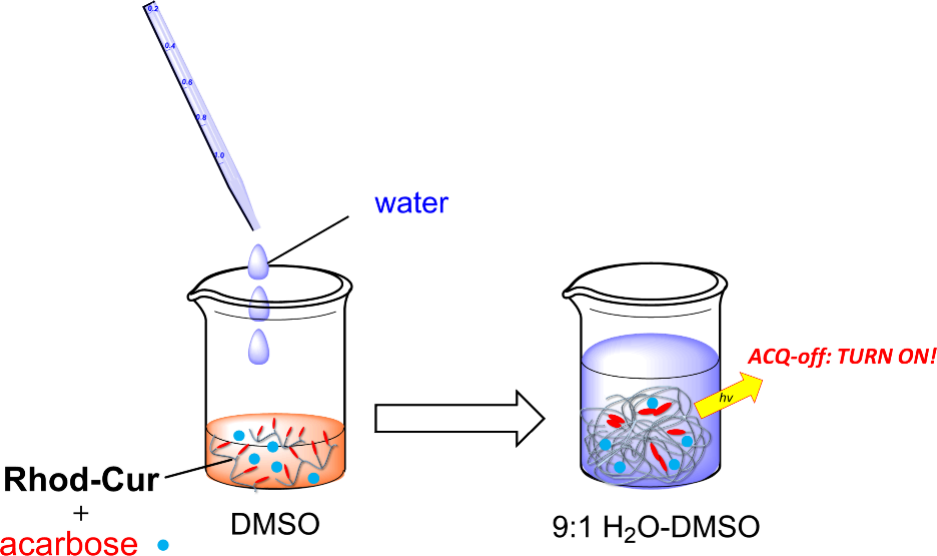

The ability to sense saccharides in aqueous media has attracted much attention in multidisciplinary sciences because the detection of ultrahigh concentrations of sugar chains associated with serious diseases could lead to further health promotion. However, there are notable challenges. In this study, a rhodamine-modified Curdlan (**Rhod-Cur**) chemosensor was synthesized that exhibited distinctive fluorescence “turn-on” responses. **Rhod-Cur** exhibited simultaneous sensitive and selective sensing of clinically useful acarbose with a good limit of detection (5 μM) from among those of the saccharides examined. The (chir)optical properties of **Rhod-Cur** were elucidated using UV/vis, fluorescence, excitation, and circular dichroism spectroscopies; lifetime measurements and morphological studies using atomic force and confocal laser scanning microscopy and dynamic light scattering techniques revealed that the fluorescence “turn-on” behavior originates from globule-to-coaggregation conversion upon insertion of the oligosaccharides in the dynamic Cur backbone.

## 1 Introduction

Recognizing and sensing higher saccharides or carbohydrates in aqueous solutions using synthetic host molecules or chemosensors are crucial from the viewpoint of the origin of life and medicinal applications.^1−14^ However, developing these chemosensors poses a notable challenge within the parameters of the current chemistry. The high-performance detection of specific oligosaccharides in physiological media has always been desired at real medical sites since real-time monitoring leads to an early diagnosis of malignant tumors, prevention of undesirable side effects from diabetes drugs, and promotes further health.^15,16^ However, simultaneous highly sensitive and selective sensing poses challenges because of the structural complexity, heavy hydration, and low concentration of oligosaccharides in human blood.^6,13,17^

Two approaches have successfully accomplished saccharide sensing in aqueous media: (1) dynamic boronate formation using the boronic acid moiety in chemosensors and saccharide diols^18−26^ and (2) supramolecular complexation in water-soluble cages and artificial lectins via noncovalent interactions, such as hydrophobic effects and CH−π interactions.^27−39^ These systems are based on the lock-and-key model, a rigid recognition method that is inherently limited by virtue of the fact that the recognition pocket should be gradually expanded/extended to precisely fit the higher saccharide analogs, accounting for their size and shape.^13^ In contrast to such nonflexible systems, one can mimic the smart nature strategy adopted by dynamic and/or induced-fit approaches.^13^ For example, lectins ingeniously distribute the multiple cooperativities of highly ordered hydrogen bonds and CH−π interactions to precisely recognize sugar chains.^40^

**Figure 1:**
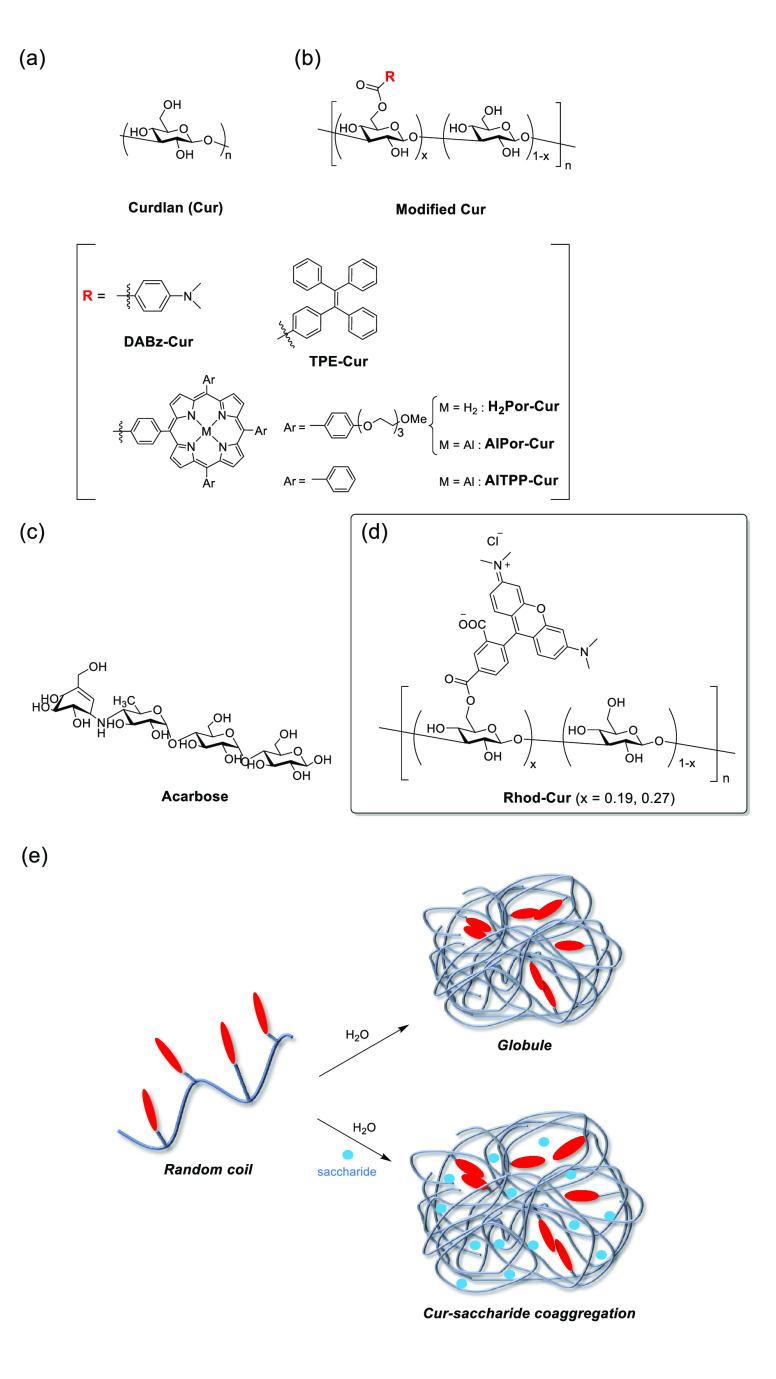
Chemical structures of (a) native curdlan (Cur), (b) modified Cur, (c) acarbose, and (d) **Rhod-Cur**. (e) Schematic illustration of the dynamic morphological changes of modified Cur via random coil-to-globule (top) and -Cur-saccharide coaggregation (bottom) conversion.

Thus, far, we have focused on the polysaccharide curdlan (Cur, Figure 1a), which is believed to function as a dynamic and induced-fit-type chemosensor.^41^ Cur is a glucan composed of β-(1,3)-linked d-glucose units that are linearly connected (without branching glucose moieties). Cur undergoes reversible renaturing/denaturing upon simply changing the solvent, a feature crucial for saccharide sensing.^42−44^ In DMSO, Cur is randomly coiled and dynamically changes to a triple helix in aqueous solutions; this behavior suggests that a target oligosaccharide can penetrate the Cur string to spread the dynamic hydrogen-bonding networks during the renaturing conversion. In 2010, we discovered that modified Cur, which has 4-dimethylaminobenzoate (DABz) appended as a reporter (Figure 1b), could trap tetrasaccharide acarbose, thus accomplishing a simultaneously sensitive and selective sensing of oligosaccharide in aqueous media.^41^

Acarbose (Figure 1c) is globally used to treat type-2 diabetes and obesity; despite its widespread use, there are associated side effects and real-time monitoring of its concentration in blood is highly desirable.^45^ When changing the DABz report for circular dichroism (CD) detection to porphyrin (Por) appendances, the limit of detection (LOD) improved from 10 mM for **DABz-Cur**,^41^ 5 mM for **H**_**2**_**Por-Cur**,^46^ 200 μM for **AlPor-Cur**,^46^ and then to 100 μM for **AlTPP-Cur**(47) (see the structures of modified Curs in Figure 1b). Morphological investigation studies on Por-Curs revealed that Cur-saccharide coaggregation induced by oligosaccharide insertion into the dynamic Cur string plays a significant role; the globule expands from its original state to that of the coaggregated form upon saccharide addition (Figure 1e).^46,47^ Based on the globular expansion, we realized that CD and fluorescent reporters can be used to elucidate the structural (morphological) changes of modified Curs upon saccharide addition. Furthermore, aggregation-induced emission (AIE)-based tetraphenylethylene (TPE) was selected because it is the first fluorophore prototype for modified Cur.^48,49^ We reported that the original globule of **TPE-Cur** initially emits strongly and its fluorescence intensity gradually decreases upon steady addition of saccharide, yielding an appreciable LOD of 5 μM.^48^ The decrease in fluorescence is consistent with the expansion of globular aggregates via globule-to-coaggregation conversion. Therefore, this finding inspired us to investigate the possibility of a “turn-on” fluorescence reporter for smart Cur chemosensors.

In this study, we harnessed aggregation-induced quenching (ACQ) behavior instead of AIE to synthesize a Cur chemosensor that exhibits turn-on fluorescence signaling. ACQ-type rhodamine appeared to be suited to this purpose because of the chemical and photophysical properties of its fluorophore, adjustable solubility in aqueous media, and good photophysical performance. Herein, we report the first example of turn-on fluorescence signaling for oligosaccharide sensing using **Rhod-Cur** in aqueous media (Figure 1d). The comparative studies discussed herein provide a basis for the creation of ultrahigh-detectable oligosaccharide chemosensors.

## 2 Experimental Section

### 2.1 Instruments

^1^H NMR (400 MHz) and ^13^C NMR (100, 125, and 150 MHz) spectra were recorded using a JNM-ESC400, ECX-500, or Bruker AVANCE III spectrometer. UV/vis, fluorescence, and CD spectra were measured in a quartz cell (10 mm path length) using a JASCO V-650 or V-560, JASCO FP-8500, or J-720WI spectrometer; all the instruments were equipped with temperature controllers. Dynamic light scattering (DLS) experiments were performed using an Otsuka ELSZ-2 instrument. Atomic force microscopy (AFM) images were obtained using a Shimadzu SPM-9600 or -9700HT microscope. Confocal laser scanning microscopy (CLSM) images were obtained using an FV3000 microscope (Olympus, Tokyo, Japan). Infrared (IR) spectra were recorded on a JASCO FT/IR-4700 spectrometer. The fluorescence lifetimes were measured using a Hamamatsu Quantaurus-Tau single-photon counting system. The fluorescence quantum yields were measured using a Hamamatsu Quantaurus-QY instrument that adopts the absolute method.^50^ The solution pH was measured by using a HORIBA standard ToupH electrode (9615-10D).

### 2.2 Materials

Fluorescence-free grade DMSO, Milli-Q water, and commercially available nonaminosaccharides were used as received. Glucosamine, valienamine, and validamycin A, purchased in acid form, were neutralized by adding an appropriate amount of an aqueous KOH solution to DMSO containing **Rhod-Cur**. The number-average molecular weight (*M*_n_) and polydispersity index (PDI) of Cur used in this study (cut-Cur) were 3.8 × 10^5^ and 3.7, respectively; the native Cur in the available form (*M*_n_ = 1.4 × 10^6^ and PDI = 3.2) was cut using a catalytic amount of an acid in advance, according to a previous method.^51^

### 2.3 Spectroscopic Studies

Stock solutions of **Rhod-Cur** were prepared by dissolving the polymer fibrils in DMSO under sonication. Sample solutions of **Rhod-Cur** in 1:9 (v/v) DMSO-H_2_O were prepared; a portion of the stock DMSO solution, which contained a given amount of saccharide, was diluted with water to a desired concentration, and the resulting mixture was stirred for 10 min and then subjected to the optical/scattering examinations.

### 2.4 Atomic Force Microscopy Measurements

A 15 μL aliquot of a 1:9 (v/v) DMSO-H_2_O solution of **Rhod-Cur** was added dropwise on a mica surface, predried under a flow of N_2_, and thereafter, fully dried under high vacuum for 5 h prior to the AFM examinations.

## 3 Results and Discussion

### 3.1 Morphological Changes of **Rhod-Cur**

**Figure 2:**
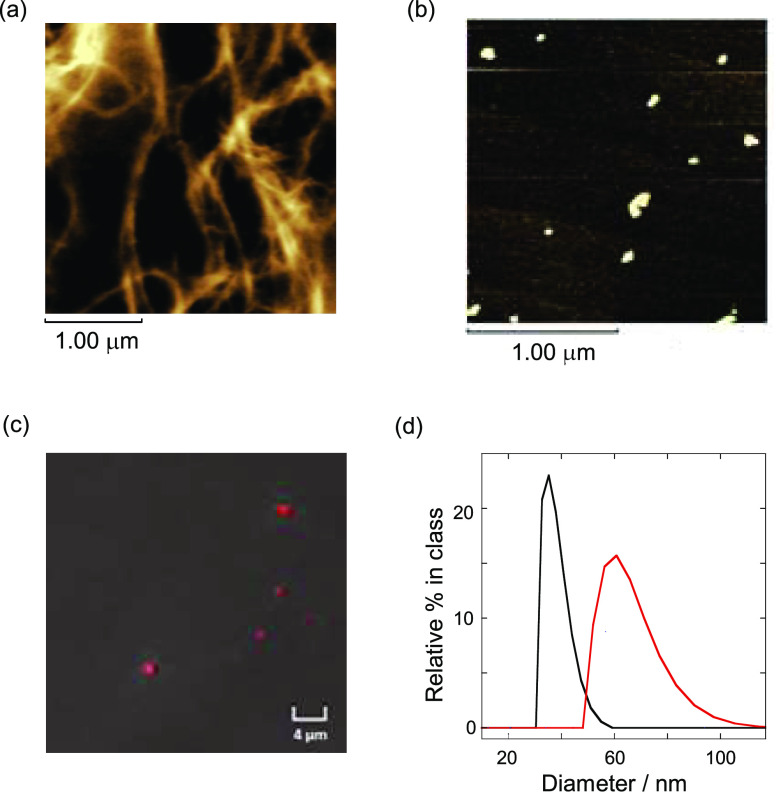
Atomic force microscopy images of (a) native Cur (100 μM in monomer units) and (b) **Rhod-Cur** (80 μM in monomer units) prepared from each 1:9 (v/v) DMSO-H_2_O solution on a mica surface. (c) Confocal laser scanning microscopic image of **Rhod-Cur** (80 μM in monomer units) in 1:9 (v/v) DMSO-H_2_O. (d) Dynamic light scattering observations of 1:9 (v/v) DMSO-H_2_O solutions of **Rhod-Cur** (15 μM in monomer units) in the absence (black) and presence of acarbose (100 μM, red).

We synthesized **Rhod-Cur** with two degrees of substitution (DS) of 0.19 and 0.27 (see the Synthesis and Characterization in the Supporting Information (SI)). The latter DS will be mainly discussed because it performed better as a sensor (Figure S3 in the SI). AFM was used to determine the morphological changes in **Rhod-Cur** to establish whether these Cur chemosensors behaved similarly to other modified Curs. For comparison, a 1:9 (v/v) DMSO-H_2_O solution of native Cur was first investigated; the solution was added dropwise to mica and thereafter dried completely. The AFM image in Figure 2a displays long and thin fibers, indicating the formation of the original triplet in the renatured state. By contrast, the AFM image of **Rhod-Cur** on a mica surface (Figure 2b) displays dotted globules as another renatured state, similar to those of other modified Curs.^46−48^ The width and height were estimated as being 79.3 ± 16.0 and 5.6 ± 1.1 nm, respectively (Figure S4 in the SI). Furthermore, rhodamine modification enabled confocal laser scanning microscopy to show clear red dotted spots in a 10% DMSO aqueous solution (Figure 2c). More importantly, DLS analysis of **Rhod-Cur** in an aqueous solution provided direct evidence of the dot-like globule structure, the hydrodynamic diameter (*d*_h_) of which can be estimated as being 37.9 ± 4.7 nm (Figure 2d, black line). These microscopic and scattering observations suggest that modifying the Cur backbone by inserting chromophores causes morphological changes from the Cur triplex to the globule, a general behavior observed in glucan chemistry; other branched glucans were also similarly observed.^49^

### 3.2 Optical Properties of **Rhod-Cur**

**Figure 3:**
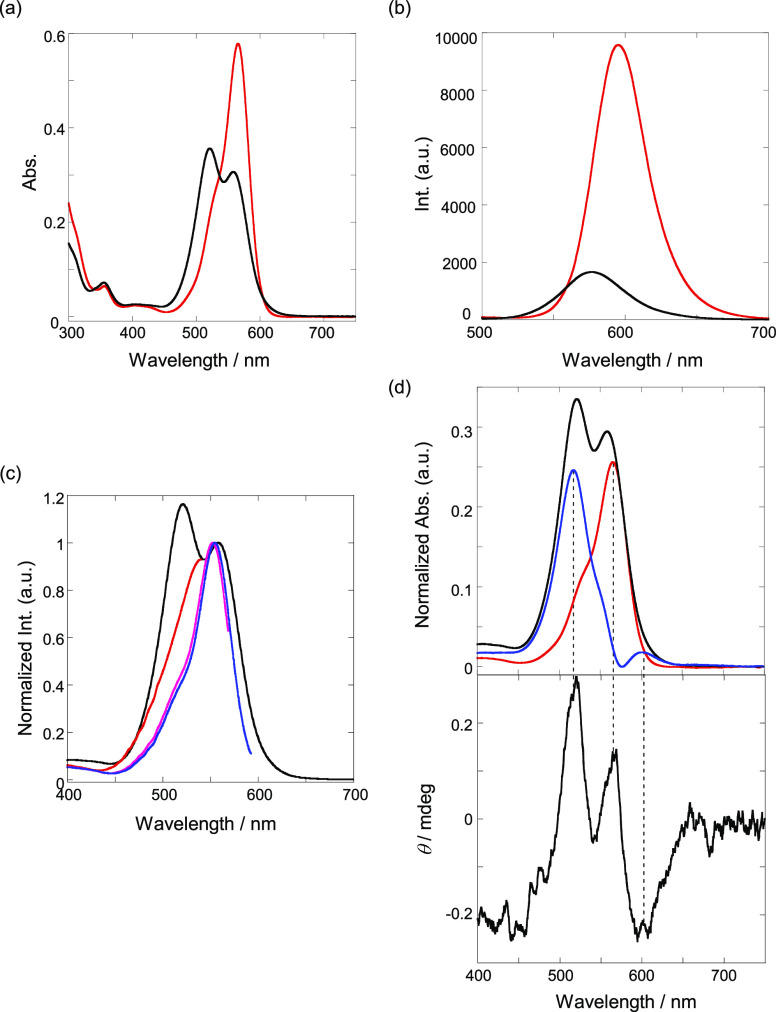
(a) UV/vis and (b) fluorescence spectra (λ_ex_ = 340 nm) of **Rhod-Cur** in DMSO (red) and 1:9 (v/v) DMSO-H_2_O (black) at 25 °C; the fluorescence intensities were corrected by the absorbances at the excitation wavelength. (c) Excitation spectra of **Rhod-Cur** in 1:9 (v/v) DMSO-H_2_O at 25 °C (UV/vis: black, 575 nm: red, 600 nm: pink, 625 nm: blue). (d) Normalized UV/vis (top) (DMSO: red, subtraction: blue) and circular dichroism (bottom) spectra of **Rhod-Cur** in 1:9 (v/v) DMSO-H_2_O (black) at 25 °C; [**Rhod-Cur**] = 21 μM in the chromophore unit.

Next, the photophysical properties of **Rhod-Cur** were investigated in DMSO and aqueous DMSO solutions to confirm that the morphological changes were related to its spectroscopic behavior (Figure 3a). In the UV/vis spectra, a sharp maximum was observed in DMSO (red line), in contrast to the suppressed and split spectral shapes observed in the 10% DMSO aqueous solution (black line). Importantly, as seen in Figure 3b, the fluorescence spectra display a quenched state intensity in the aqueous solution (black line) compared to that observed in DMSO (red line). The fluorescence quantum yields (Φ_F_) are 0.011 in 10% aqueous DMSO and 0.063 in DMSO, suggesting that the rhodamine fluorophores on the Cur backbone assemble in the globule and impart aggregate-state photophysical properties. Moreover, as shown in Figure 3c, the fluorescence excitation spectral changes exhibit a dependence on the monitoring wavelength, suggesting the formation of ground-state stack species. To elucidate the origin of the excited species, the fluorescence lifetimes were investigated in 10% DMSO aqueous solution. The lifetime decay profiles comprised those of multiple components and fit reasonably to the sum of two exponentials to afford lifetimes (τ) of 2.9 and 0.9 ns, as listed in Table 1 (the fitting results are shown in Figure S5 in the SI). The longer-lived species (2.9 ns) can be reasonably ascribed to the monomer-state rhodamine; thus, the shorter-lived species (0.9 ns) are assigned to the ground-state stacked (aggregated) species based on the promoted radiationless deactivation path (2.9 → 0.9 ns).^52^ The CD spectral analysis further aided in the precise assignment of the aggregated species. As shown in Figure 3d, the subtracted UV/vis absorption (10% DMSO (black line)–DMSO (red line) = blue line) displays two split peaks at 517 and 601 nm; the monomer peak at 565 nm is in the middle of the two DMSO peaks. The two peaks exhibit positive and negative Cotton effects, indicating that the two species are different aggregates, which may be reasonably ascribed to the *H*- and *J*-aggregates located at shorter and longer wavelengths, respectively.^52,53^ Thus, the fluorescence-quenching state based on aggregation is adopted in the original globule.

**Table 1:** Fluorescence Lifetimes of Rhod-Cur^a^

solvent	*n*^b^	τ_1_ (ns)	*A*_1_	τ_2_ (ns)	*A*_2_	χ^2^
DMSO^c^	2	2.9	0.18	0.8	0.82	1.2
10% DMSOaq.^c^	2	2.9	0.48	0.9	0.52	1.2
10% DMSOaq.^d^	2	2.9	0.47	0.9	0.53	1.2

a Fluorescence lifetime (τ_i_) and relative abundance (*A*_i_) of each excited species; λ_em_ = 625, 650 nm; [**Rhod-Cur**] = 21 μM in the chromophore unit.

b Number of components.

c Without acarbose.

d With acarbose (100 μM).

### 3.3 “Turn-On” Acarbose Sensing in Aqueous Media

**Figure 4:**
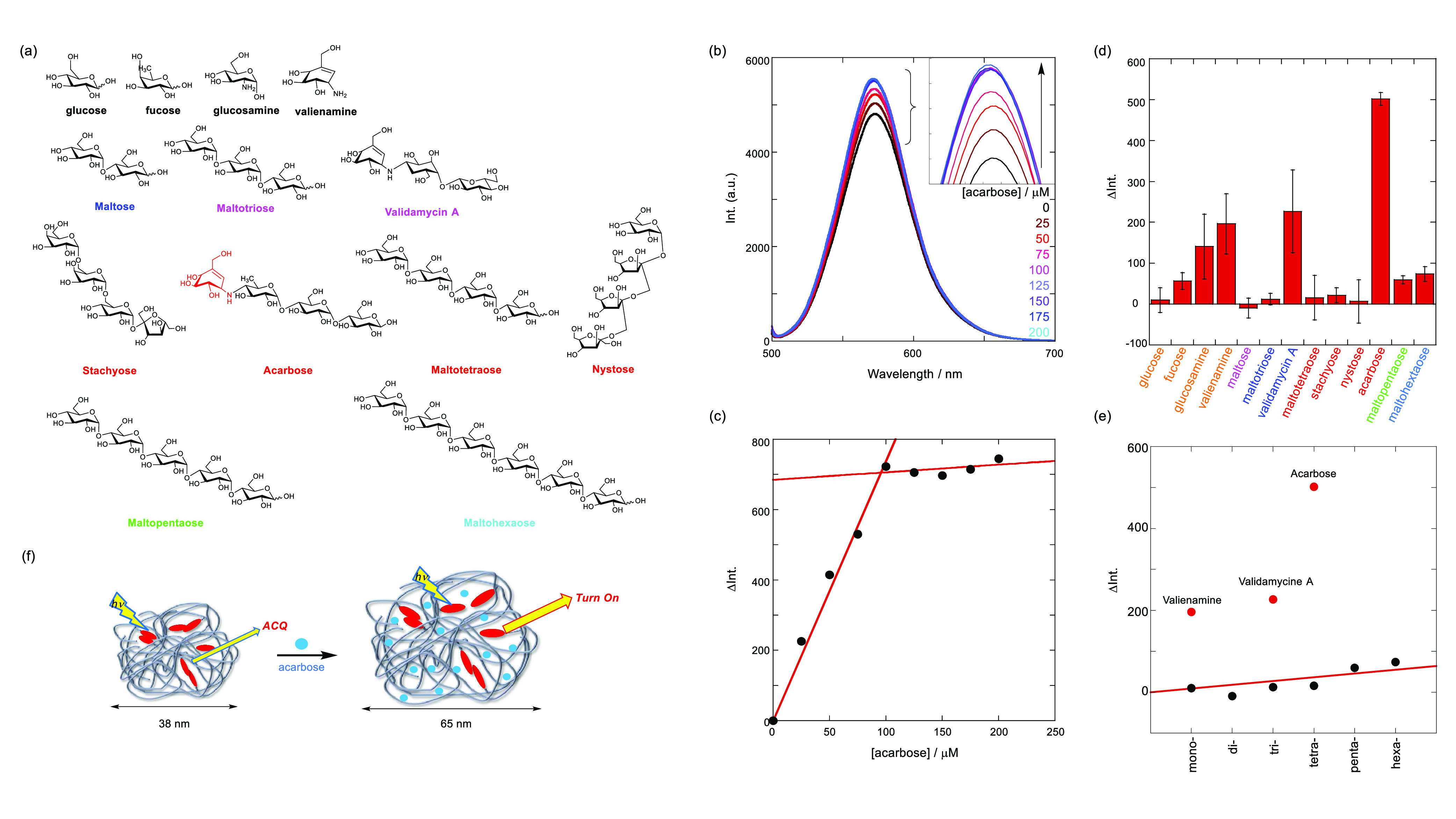
(a) Chemical structures of the saccharides examined in this study (from mono- to hexa-saccharides). (b) Fluorescence spectra (λ_ex_ 485 nm) of **Rhod-Cur** (80 μM in monomer unit) in the absence (black) and presence of acarbose (25, 50, 75, 100, 125, 150, 175, and 200 μM, colored lines) in 1:9 (v/v) DMSO-H_2_O at room temperature; the fluorescence intensities were corrected by the absorbances at the excitation wavelength. (c) ΔInt. (λ_obs_ 573 nm) data obtained from (b) were plotted as a function of the acarbose concentration; ΔInt. represents the delta fluorescence intensity; ΔInt. = 7.38 [acarbose (μM)]. (d) ΔInt. (λ_obs_ = 575 nm) of **Rhod-Cur** (80 μM in the monomer unit) induced upon complexation of the tested saccharides (100 μM). (e) ΔInt. data obtained from (d) plotted as a function of saccharide length. (f) Schematic illustration of the fluorescence “turn-on” change based on the globular expansion.

We used acarbose as a target for oligosaccharide sensing, because it can induce a wide fluorescence change (*vide infra*); the structures of all the saccharides that were tested are shown in Figure 4a. Upon the addition of acarbose (Figure 4b), the quenched-state fluorescence was recovered and displayed the “turn-on” fluorescence behavior based on saccharide-Cur coaggregation expansion. The *d*_h_ value of the coaggregation obtained using DSL measurement was 65.2 ± 10.7 nm (Figure 2d, red line), indicating that the flexible Cur string renatures to a much expanded globule upon insertion of the saccharide (Figure 4f). The morphological changes counteract a certain degree of the original aggregation states of the fluorophores, thereby causing fluorescence recovery, that is, “turn-on.”

In Figure 4c, the titration data show a good straight line up to 100 μM and thereafter reach a plateau simply because of the solubility limitation of coaggregation. Thus, in the dynamic range, the slope was estimated as a sensitivity (Δ_int_ = 7.38 [acarbose (μM)]); the sensitivity is slightly higher than that (−5.94) of the previous “turn-off” chemosensor (**TPE-Cur**).^48^ Moreover, the LOD value was also estimated as 5 μM (see the data and LOD definition in Figure S6 in the SI), which is equal to that of **TPE-Cur**.^48^ It is noteworthy that **Rhod-Cur** enables ultrahigh detection of the medically useful acarbose with the lowest LOD based on the fluorescence “turn-on” behavior.

### 3.4 Mechanistic Investigations on Selectivity, “Turn-On”, and Factors Controlling Inherent Oligosaccharide Sensing

**Figure 5:**
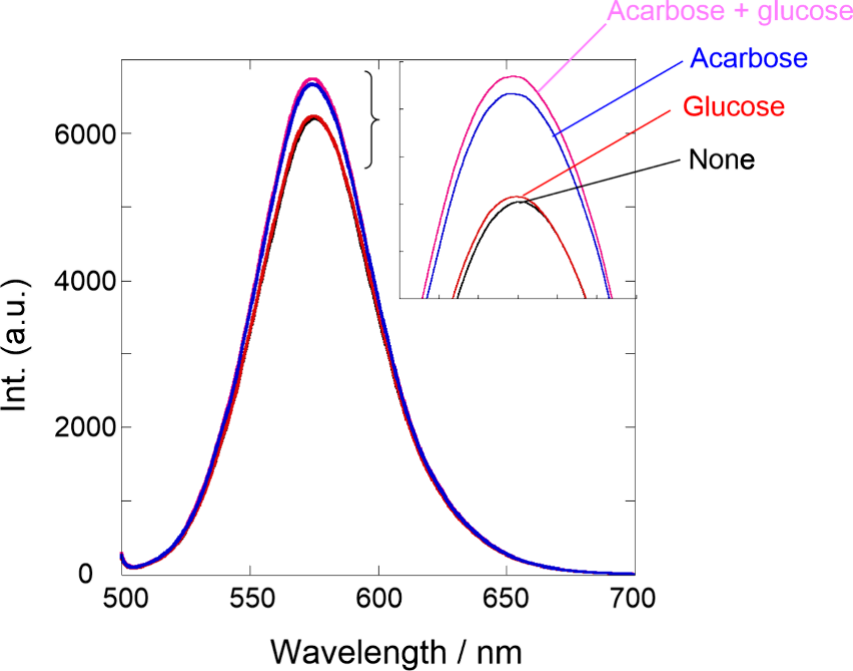
Fluorescence spectra (λ_ex_ = 485 nm) of **Rhod-Cur** (80 μM in monomer unit) in the absence (black) and presence of glucose (5 mM, red), acarbose (100 μM, blue), and glucose/acarbose (5 mM/100 μM, pink) in 1:9 (v/v) DMSO-H_2_O at room temperature; the fluorescence intensities were corrected by the absorbances at the excitation wavelength.

To elucidate the selectivity of **Rhod-Cur**, we investigated the “turn-on” responses to various mono- and hexa-saccharides (the titration data are shown in Figures S7–9 in the SI). As shown in Figure 4d, the fluorescence intensities of **Rhod-Cur** increase for all of the saccharides tested upon interaction, indicating the formation of saccharide-Cur-expanded coaggregates. This expansion was proved by the DLS data (*vide supra*). Furthermore, as shown in Table 1, the lifetime data in the absence and presence of acarbose were almost the same and did not distinguish each other, simply because of their insensitivity. However, this means the significance of the “turn-on” detection by reading out fluorescence intensity. Notably, there is a large fluorescence enhancement upon interaction with acarbose and its derivative, validamycin A, thereby indicating that the acarbose skeleton strongly interacts with the dynamic **Rhod-Cur** backbone. Another noteworthy observation is that the fluorescence responses correlate with the saccharide length (Figure 4e), indicating that the polysaccharide–saccharide interactions play an important role. Among the saccharides tested, acarbose and validamycin A have a valienamine structure (red moiety in Figure 4a), which is most probably the origin of the high sensitivity toward coaggregation formed by **Rhod-Cur**. The fluorescence change exhibited by valienamine upon interaction is larger than that of the other monosaccharides. The specificity of valienamine may be reasonably accounted for by the amine donor moiety as compared with a similar glucosamine with an amine group. To further investigate the significance of amino groups, we tested using the hydrochloric form of glucosamine (glucosamine·HCl) instead of the neutral glucosamine. As shown in Figure S10 in the SI, the addition of glucosamine·HCl to 10% DMSO aqueous containing **Rhod-Cur** did not show any significant fluorescent augmentation, indicating the there is no spontaneous interaction of cationic amino group with the Cur OH group simply because the proton donor character in N^+^–H decreases rather than that in the neutral N–H group. Thus, hydrogen-bonding interactions between the Cur OH group and the N–H moiety play a significant role during the interaction between **Rhod-Cur** and the saccharide. Therefore, the distinctive “turn-on” fluorescence response for acarbose (valienamine + maltotriose), compared with those of the other saccharides examined, for example, validamycin A (valienamine + maltose), can be accounted for by mutual interactions based on the hydrogen-bonding donor valienamine skeleton and the longer saccharide chain function in the dynamic Cur backbone. Finally, we further investigated the competition experiment using acarbose and glucose. As shown in Figure 5, the excess amount of glucose (5 mM) did not alter fluorescence intensity. By contrast, the mixture of acarbose (100 μM) and glucose (5 mM) augmented the fluorescence intensity that matched the value obtained in the addition of only acarbose. This further supports the distinctive strong interaction of tetrasaccharide acarbose rather than monosaccharide glucose.

## 4 Conclusions

In conclusion, we developed a fluorescence “turn-on” oligosaccharide chemosensor, **Rhod-Cur**, that can trap the clinical drug acarbose; the sensor exhibited a low LOD of 5 μM. The simultaneous sensitive and selective sensing of acarbose, accomplished herein, which is based on “turn-on” signaling, may be extremely important for eye-catch detection. Therefore, this study could serve as a general guide for the development of ultrahigh-detectable, readable, “turn-on” oligosaccharide chemosensors that can precisely sense diverse saccharides and sugar chains related to tumor markers.

## Supporting Information Available

Synthesis and characterization, acarbose sensing using **Rhod-Cur**_**0.19**_, AFM images and line profiles, fluorescence decays, determination of LOD, and titrations for standard deviation (PDF)
