# Supplementary material for: Fluorophore-Probed Curdlan Polysaccharide Chemosensor: “Turn-On” Oligosaccharide Sensing in Aqueous Media
Source: ACS Omega. 2024 May 13;9(20):22345–51. doi: 10.1021/acsomega.4c01786 (PMC11112708; doi:10.1021/acsomega.4c01786)
Supplement: Supplementary file 1 — ao4c01786_si_001.pdf [file ao4c01786_si_001.pdf]

Supporting Information

for

Fluorophore-Probed Curdlan Polysaccharide Chemosensor: "Turn-On" Oligosaccharide Sensing in Aqueous Media

Masahiro Norikuni,† Yumiko Hori,‡ Munenori Numata,§ Michiya Matsusaki,† Toshiyuki Kida,† and Gaku Fukuhara*,‡

† *Department of Applied Chemistry, Osaka University, 2-1 Yamada-oka, Suita 565-0871, Japan*

‡ *Department of Chemistry, Tokyo Institute of Technology, 2-12-1 Ookayama, Meguro-ku, Tokyo 152-8551, Japan*

§ *Department of Biomolecular Chemistry, Graduate School of Life and Environmental Sciences, Kyoto Prefectural University, Shimogamo, Sakyo-ku, Kyoto 606-8522, Japan*

E-mail: gaku@chem.titech.ac.jp

Synthesis and Characterization

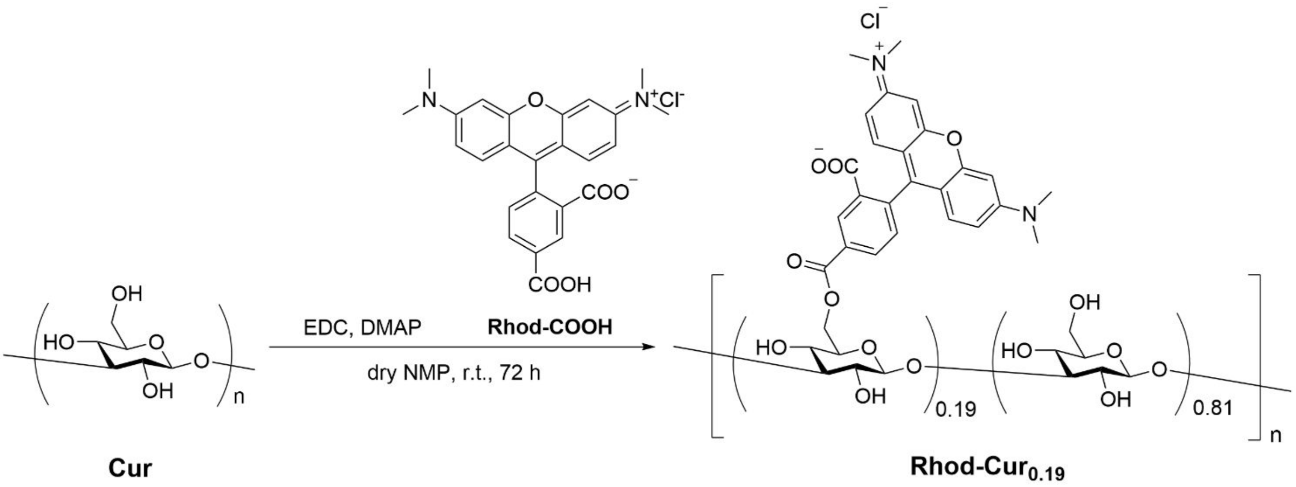

Rhod-Cur$_{0.19}$ (DS = 0.19). In a three necked flask, cut-Cur (103 mg, 0.632 mmol in glucose units) was added to dry *N*-methyl-2-pyrrolidinone (NMP, 20 mL) and the resulting highly viscous solution was heated to 100 °C and stirred for 12 h under an N_2 atmosphere. After cooling to room temperature, **Rhod-COOH**[1] (220 mg, 0.514 mmol) was dissolved in the NMP solution. Subsequently, 1-ethyl-3-(3-dimethylaminopropyl)carbodiimide hydrochloride (EDC) (984 mg, 5.14 mmol) and *N*,*N*-dimethyl-4-aminopyridine (DMAP) (627 mg, 5.12 mmol) were added to the NMP solution and the resulting solution was stirred for 3 d. To promote the reaction, extra EDC (987 mg, 5.15 mmol) and DMAP (628 mg, 0.93 mmol) were added every 24 h (three times in total). After 3 d, the reaction mixture was slowly poured into methanol (400 mL) to give a purple precipitate, which was collected, triturated, washed with methanol (400 mL), and thereafter dried under high vacuum to afford **Rhod-Cur** in 18% yield (28 mg, 0.114 mmol monomer units) as a purple solid. ^{1}H NMR (600 MHz, DMSO-d_6) δ_H 8.72 (s, 1H, H_1'), 8.43 (s, 1H, H_2'), 8.00 (s, 1H, H_3'), 7.65 (s, 2H, H_4'), 7.34–6.56 (m, 4H, $H_5'H_6'$), 4.52 (s, H_1), 3.57 (br, H_3H_6), 3.21 (m, $H_2H_4H_5$); ^{13}C NMR (125 MHz, DMSO-d_6) no peak were found due to poor solubility; IR ν3350, 2927, 2363, 2322, 1650, 1596, 1491, 1407, 1346, 1252, 1097, 1000 cm^{-1}.

Determination of the degree of substituent (DS) for Rhod-Cur. The DS (x) of **Rhod-Cur** was

determined by UV/vis spectroscopy using a DMSO solution of **Rhod-COOH** of a known concentration as a reference compound. Thus, the DS (x) of **Rhod-Cur** was calculated as 0.19 from the following equation: Abs (**Rhod-Cur**) = ε (**Rhod-COOH**) × c (concentration of **Rhod-Cur** in chromophore units) × l (path length).

[1] Kvach, M. V.; Stepanova, I. A.; Prokhorenko, I. A.; Stupak, A. P.; Bolibrukh, D. A.; Korshun, V. A.; Shmanai, V. V. *Bioconjugate Chem.* **2009**, *20*, 1673–1682.

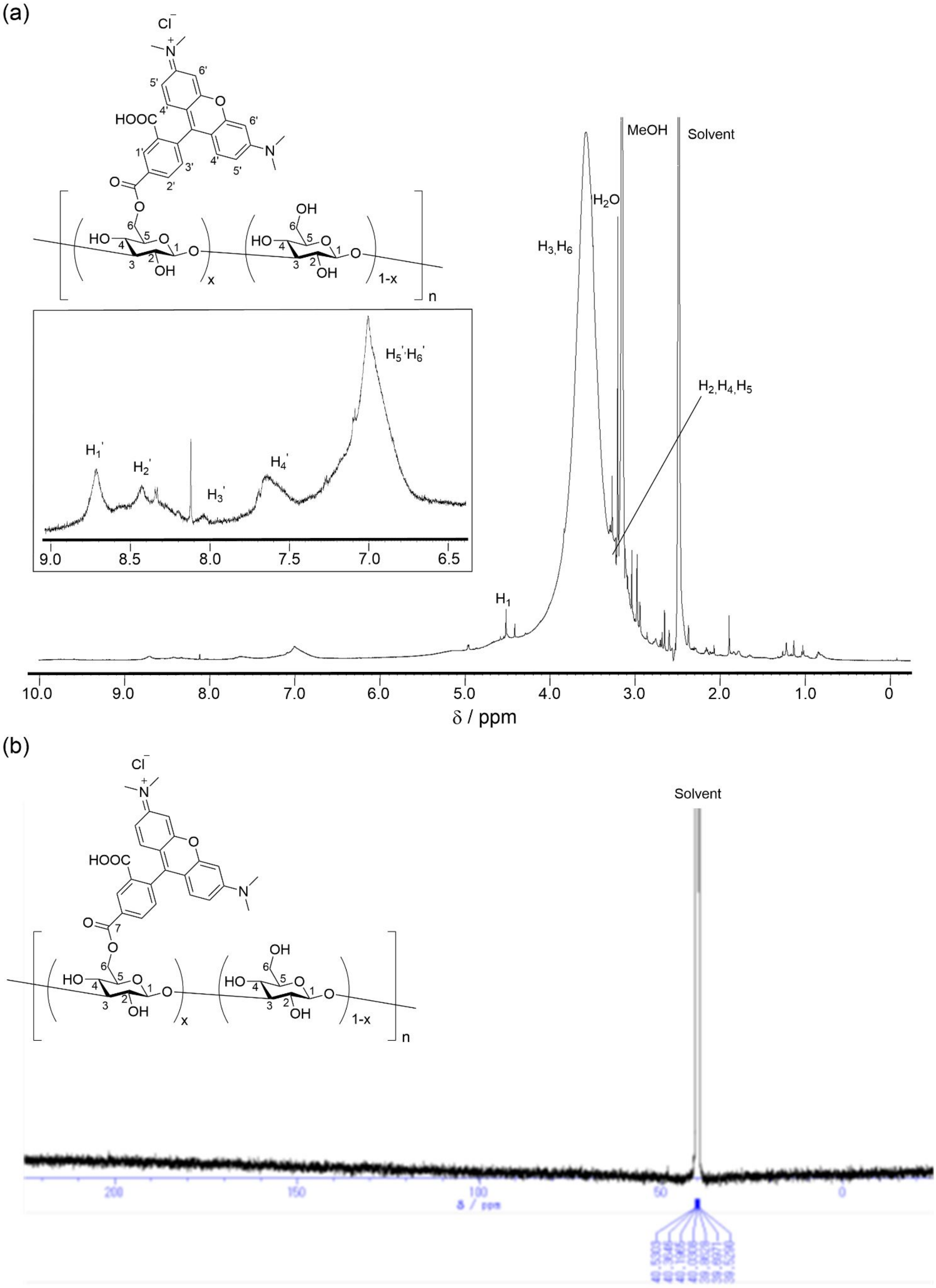

Figure S1. (a) ^{1}H (600 MHz) and (b) ^{13}C NMR (125 MHz) spectra of **Rhod-Cur$_{0.19}$** in DMSO-d_6 at room temperature.

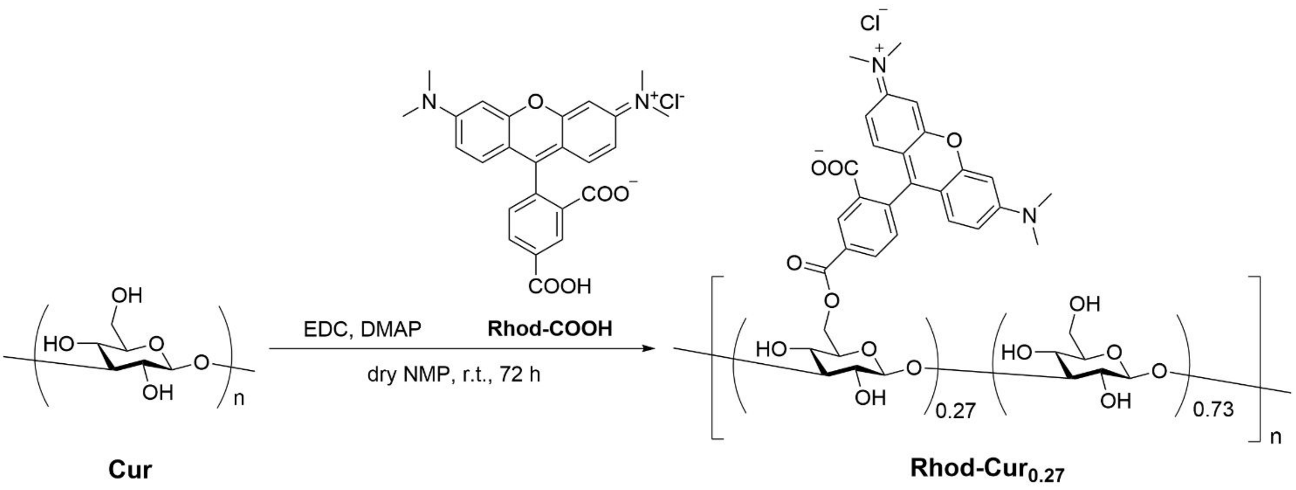

Rhod-Cur$_{0.27}$ (DS = 0.27). In a three necked flask, cut-Cur (104 mg, 0.641 mmol in glucose units) was added to NMP (6 mL) and the resulting highly viscous solution was heated to 100 °C and stirred overnight under an N_2 atmosphere. After cooling to room temperature, **Rhod-COOH** (132 mg, 0.308 mmol) was dissolved in the NMP solution. Thereafter, EDC (605 mg, 3.16 mmol) and DMAP (378 mg, 3.09 mmol) were added to the NMP solution and the resulting solution stirred for 3 d. To promote the reaction, extra EDC (599 mg, 3.12 mmol) and DMAP (378 mg, 3.09 mmol) were added every 24 h (three times in total). After 3 d, the reaction mixture was slowly poured into methanol (400 mL) to give a purple precipitate, which was collected, triturated, washed with methanol (400 mL), and thereafter dried under high vacuum to afford **Rhod-Cur** in 68% yield (98 mg, 0.436 mmol monomer units) as a purple solid. ^{1}H NMR (600 MHz, DMSO-*d*$_6$) δ_H 8.75 (s, 1H, H_1'), 8.46 (s, 1H, H_2'), 8.07 (s, 1H, H_3'), 7.66 (s, 2H, H_4'), 7.06–7.00 (m, 4H, $H_5'H_6'$), 4.51 (s, H_1), 3.64 (br, H_3H_6), 3.28 (m, $H_2H_4H_5$); ^{13}C NMR (150 MHz, DMSO-*d*$_6$) δ_C 174.2 (C_7), 103.5 (C_1), 86.7 (C_3), 76.8 (C_5), 73.3 (C_2), 68.9 (C_4), 61.4 (C_6); IR ν3308, 2873, 2359, 2330, 1715, 1647, 1596, 1336, 1252, 1028 cm^{-1}.

Determination of degree of substitution for Rhod-Cur. The DS (x) of **Rhod-Cur** was determined by

UV/vis spectroscopy using a DMSO solution of **Rhod-COOH** of a known concentration as a reference compound. Thus, the DS (x) of **Rhod-Cur** was calculated as 0.27 from the following equation: Abs (**Rhod-Cur**) = ε (**Rhod-COOH**) × c (concentration of **Rhod-Cur** in chromophore units) × l (path length).

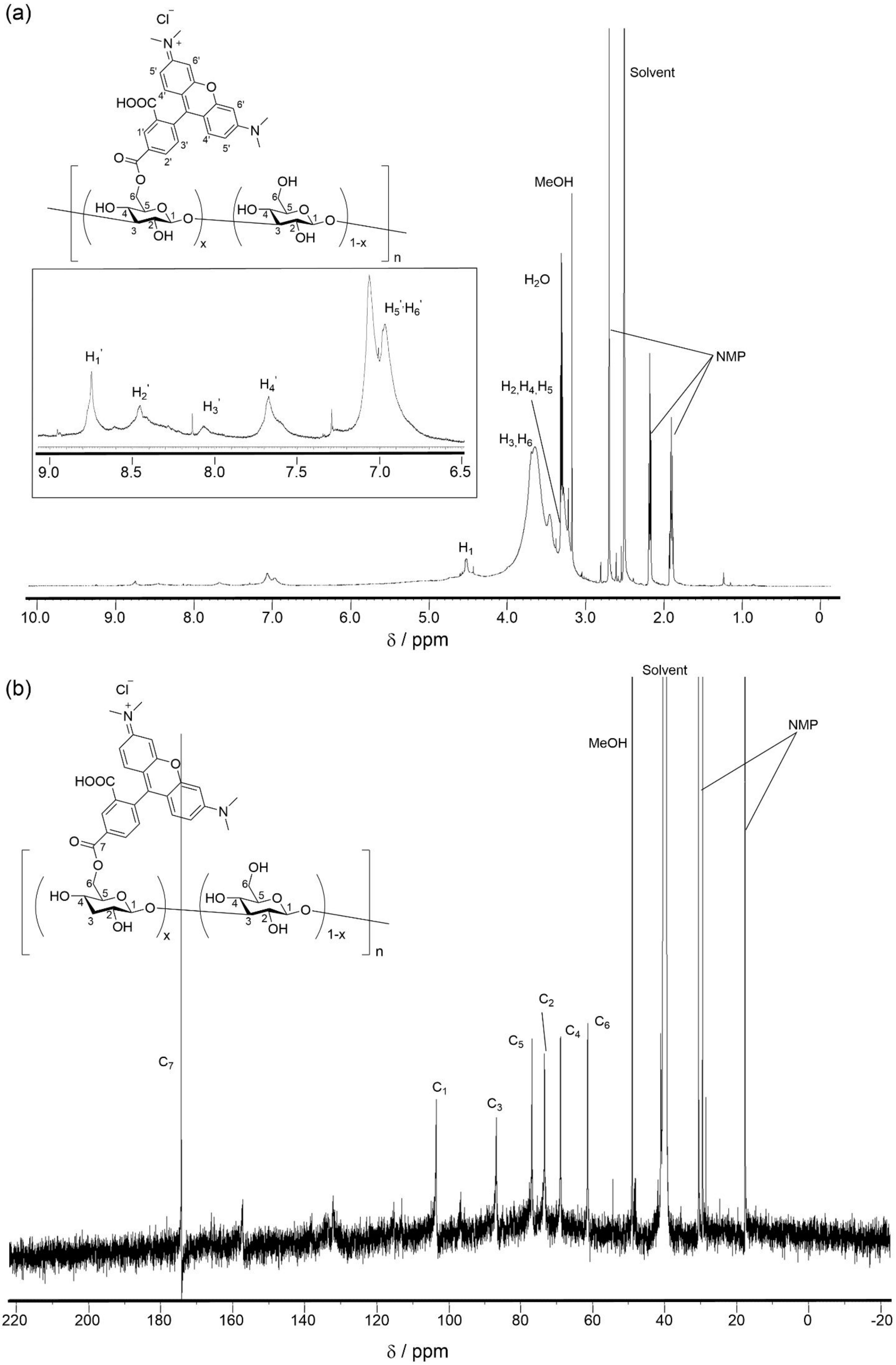

Figure S2. (a) ^{1}H (600 MHz) and (b) ^{13}C NMR (150 MHz) spectra of **Rhod-Cur$_{0.27}$** in DMSO-d_6 at room temperature.

Acarbose Sensing Using Rhod-Cur$_{0.19}$

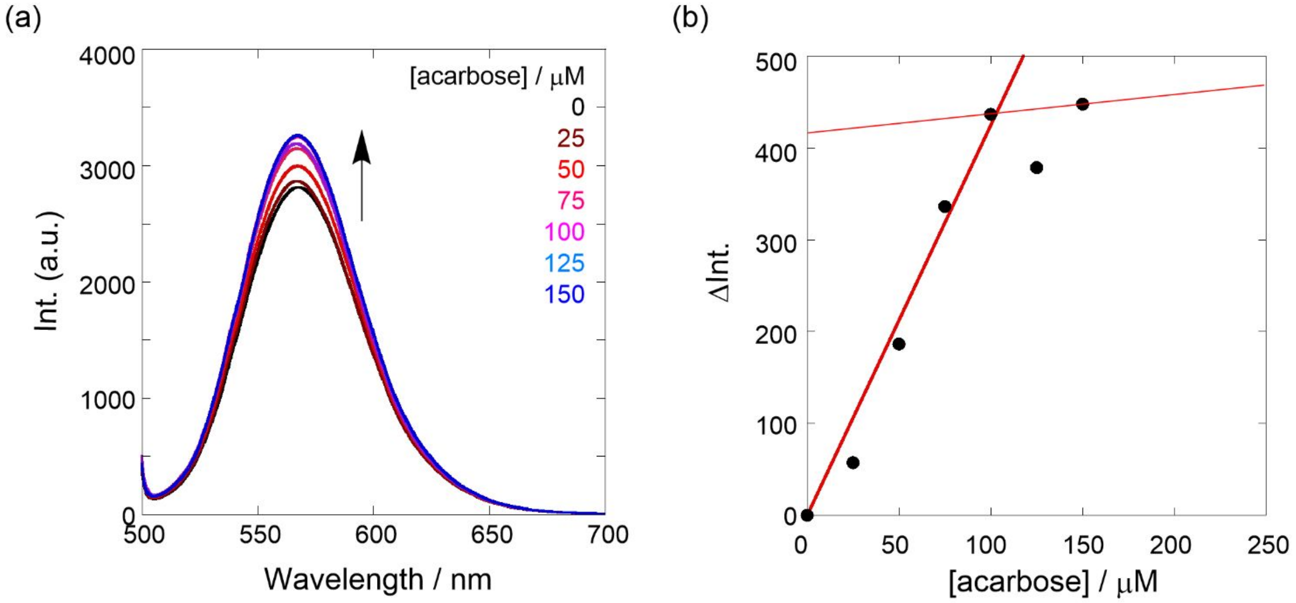

Figure S3. (a) Fluorescence spectra (λ_{ex} 485 nm) of **Rhod-Cur$_{0.19}$** (13 μM in monomer units) in the absence (black) and presence of acarbose (25, 50, 75, 100, 125, and 150 μM, colored lines) in 1:9 (v/v) DMSO-H_2O at room temperature; the fluorescence intensities were corrected by the absorbances at the excitation wavelength. (b) ΔInt. (λ_{obs} 567 nm) data obtained from (a) were plotted as a function of the acarbose concentration; ΔInt. = 4.25 [acarbose (μM)].

Atomic Force Microscopy Images of Rhod-Cur

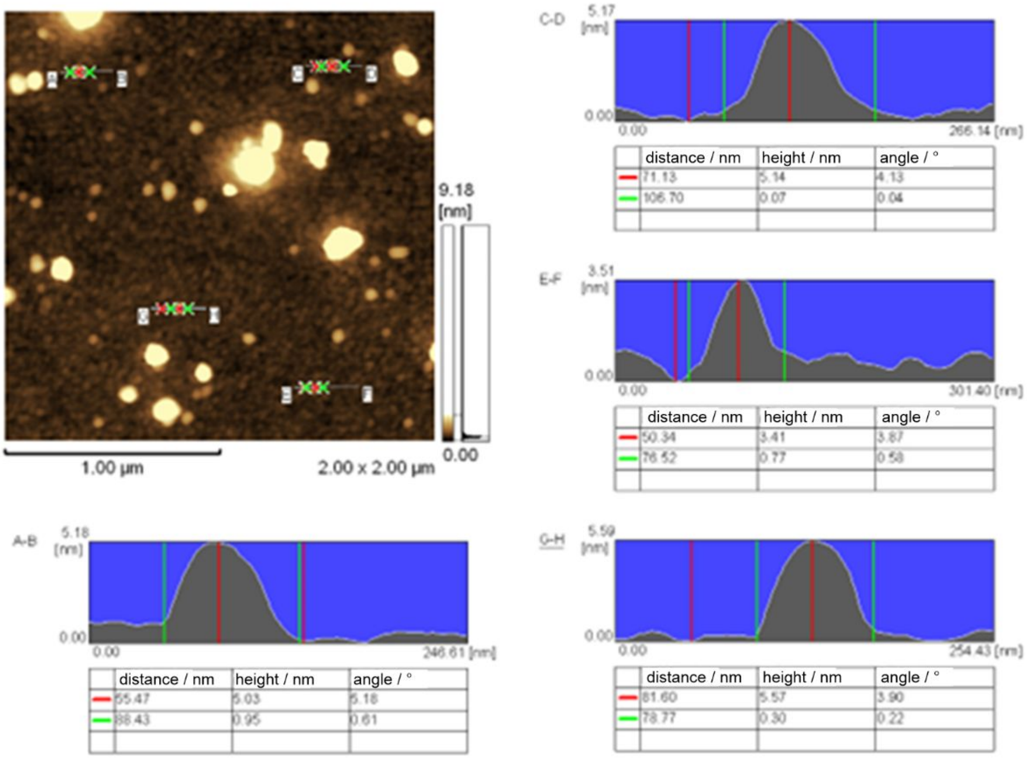

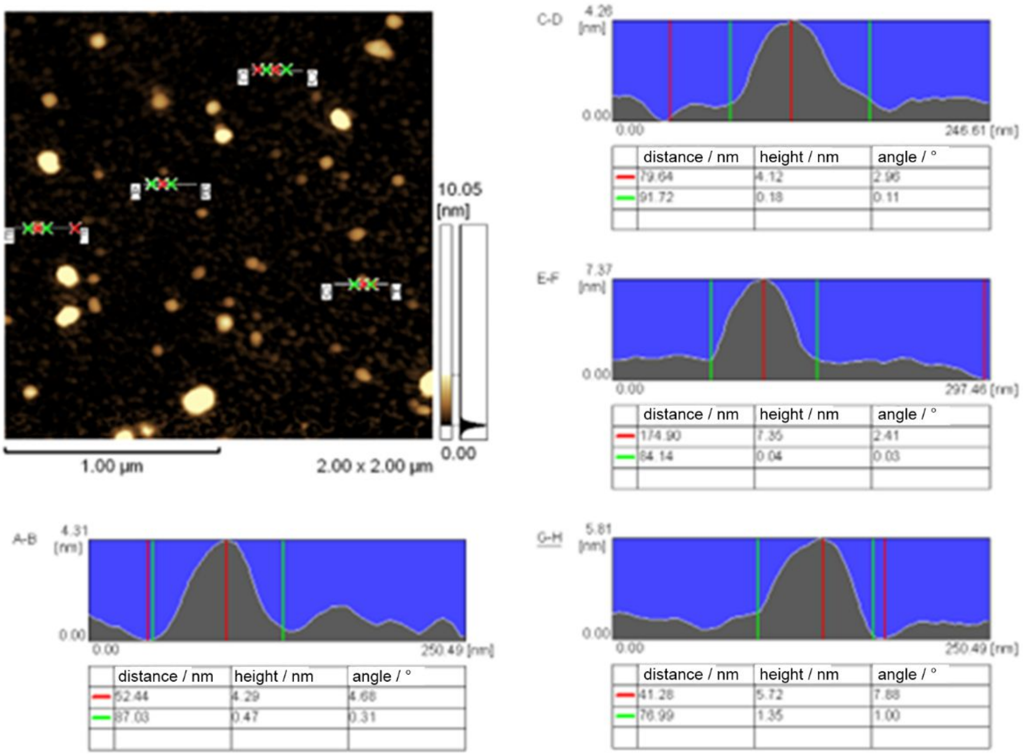

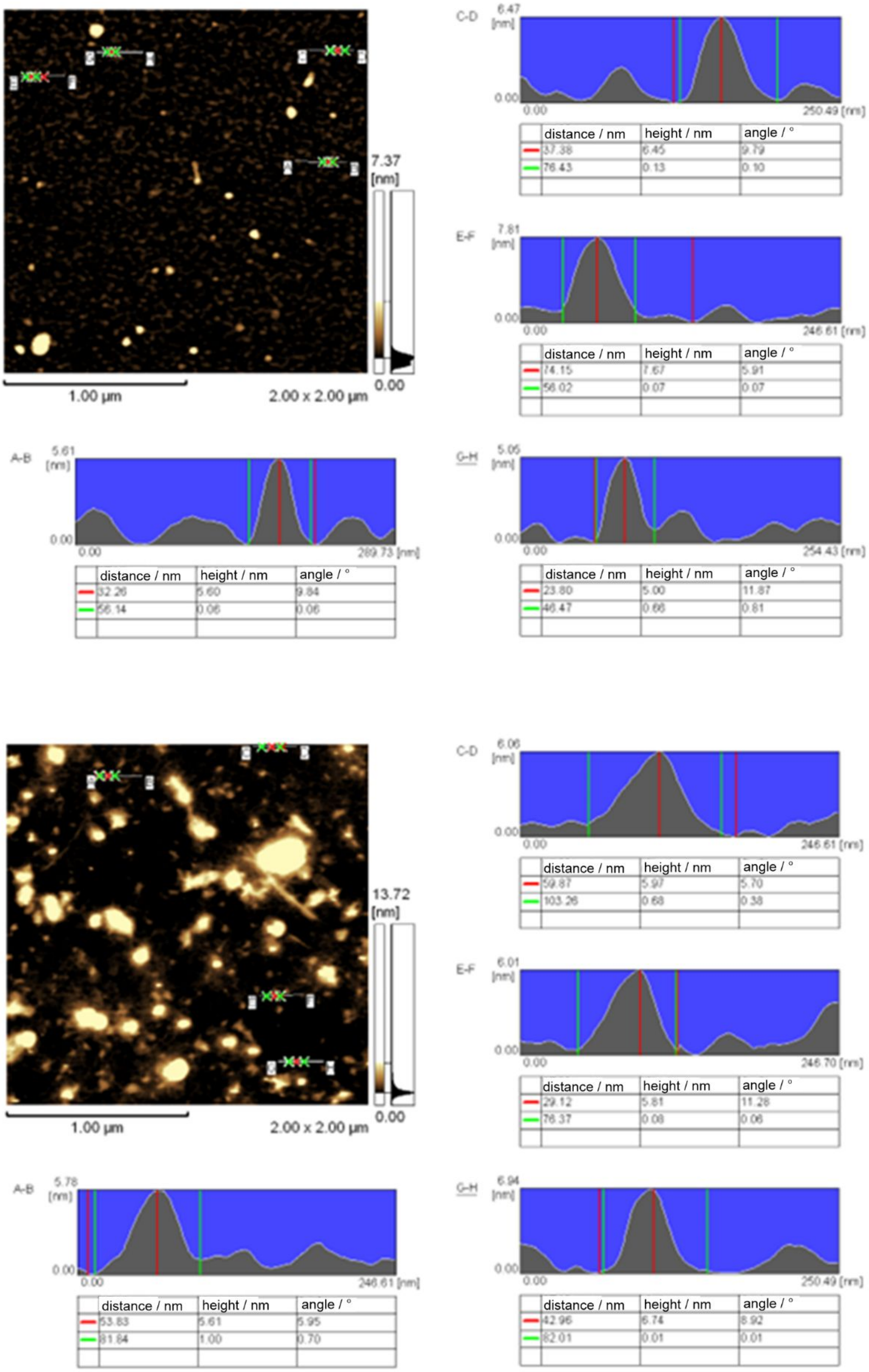

Figure S4. Atomic force microscopy images and line profiles of **Rhod-Cur$_{0.27}$** on a mica surface prepared from 1:9 (v/v) DMSO-H_2O solution.

Fluorescence Decays of Rhod-Cur$_{0.27}$

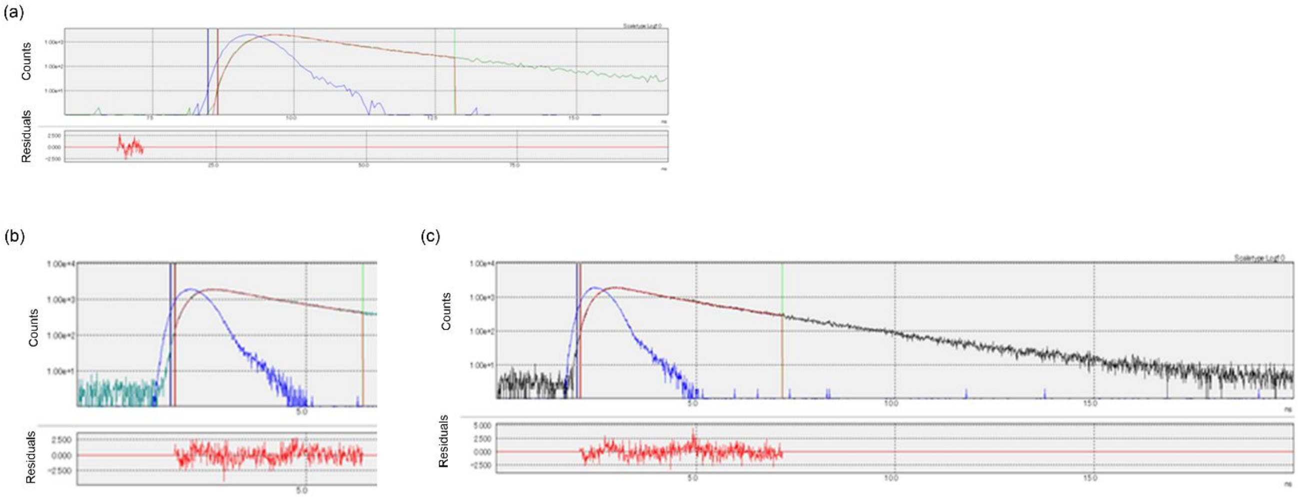

Figure S5. Time-correlated fluorescence decays of **Rhod-Cur$_{0.27}$** (21 μM in chromophore unit) in (a) DMSO (λ_{obs} 650 nm) and (b) 1:9 (v/v) DMSO-H_2O (λ_{obs} 625 nm) without acarbose and (c) 1:9 (v/v) DMSO-H_2O (λ_{obs} 625 nm) with acarbose (100 μM) at room temperature. The colored, red, and blue lines represent the fluorescence decay, fitting result, and the instrument response function, respectively.

Determination of Limit of Detection

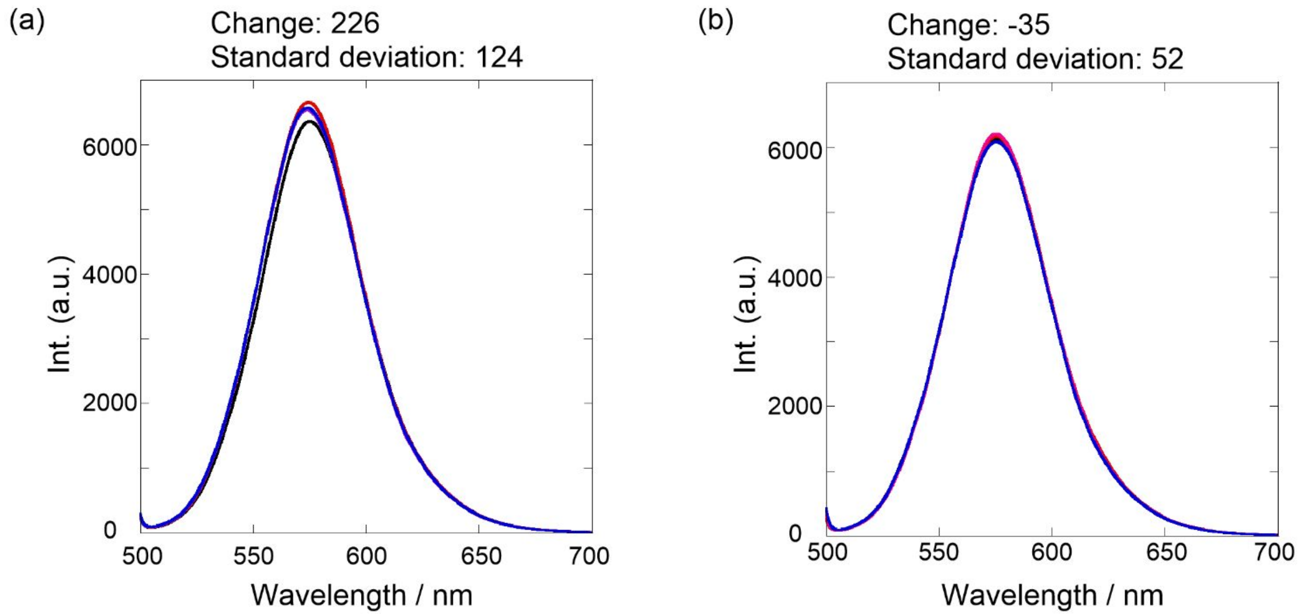

Figure S6. Fluorescence spectra (λ_{ex} 485 nm) of **Rhod-Cur$_{0.27}$** (80 μM in monomer units) in the absence (black) and presence of 5 μM (a) and 4 μM (b) acarbose in 1:9 (v/v) DMSO-H_2O at room temperature; the fluorescence intensities were corrected by the absorbances at the excitation wavelength. Three measurements were performed for each sample (1st run; red, 2nd run; blue, and 3rd run; green).

Limit of detection definition: The titration concentration at which the average fluorescence change exceeded the standard deviation was determined as the LOD: 5 μM (226 > 124) and not 4 μM (|-35| < 52).

Titrations of Saccharides to Measure Standard Deviations

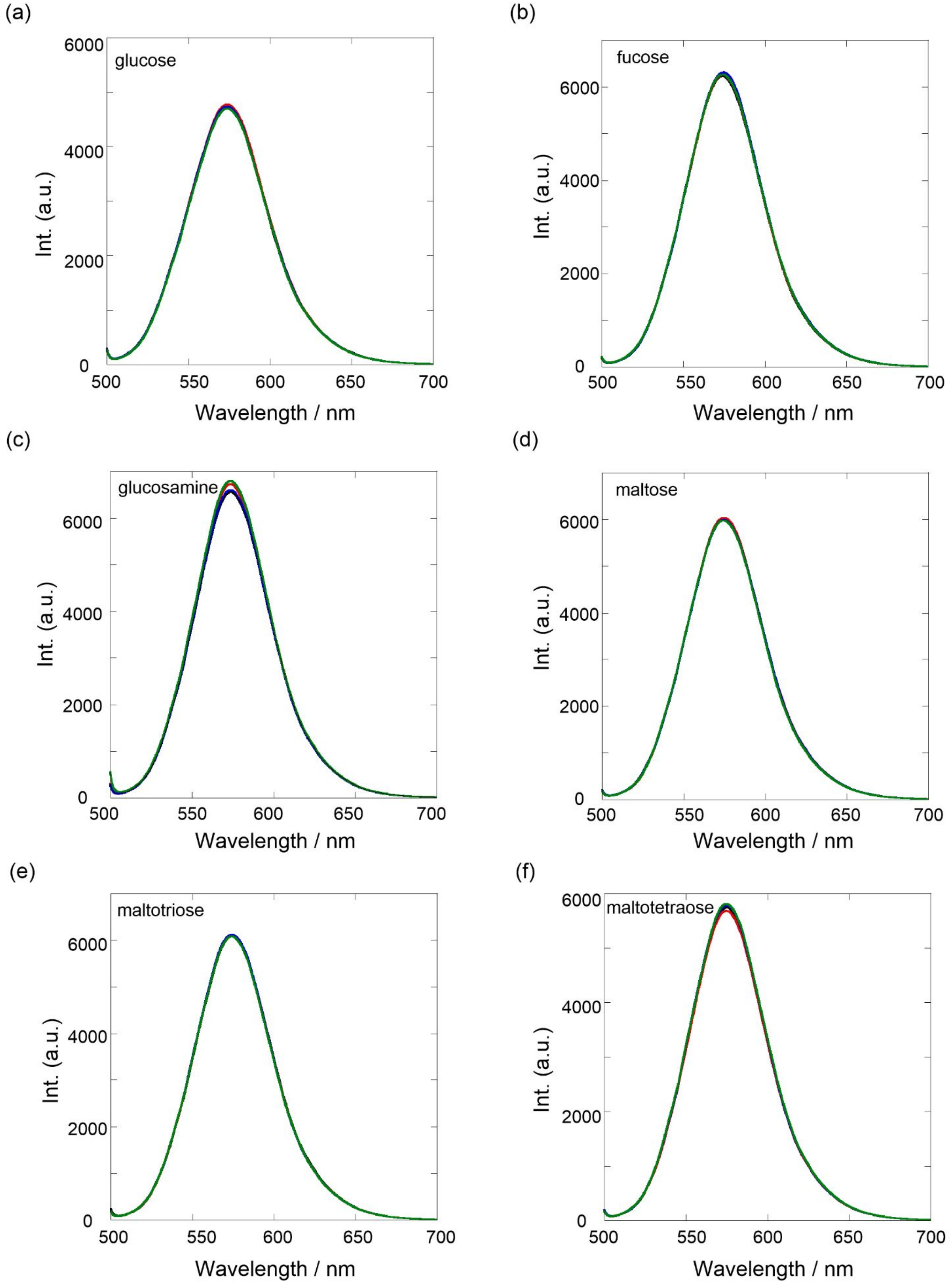

Figure S7. Fluorescence spectra (λ_{ex} 485 nm) of **Rhod-Cur$_{0.27}$** (78 μM in monomer units) in the absence (black) and presence of 100 μM glucose (a), fucose (b), glucosamine (c), maltose (d), maltotriose (e), and maltotetraose (f) in 1:9 (v/v) DMSO-H_2O at room temperature; the fluorescence intensities were corrected by the absorbances at the excitation wavelength. Three measurements were performed for each sample (1st run; red, 2nd run; blue, and 3rd run; green).

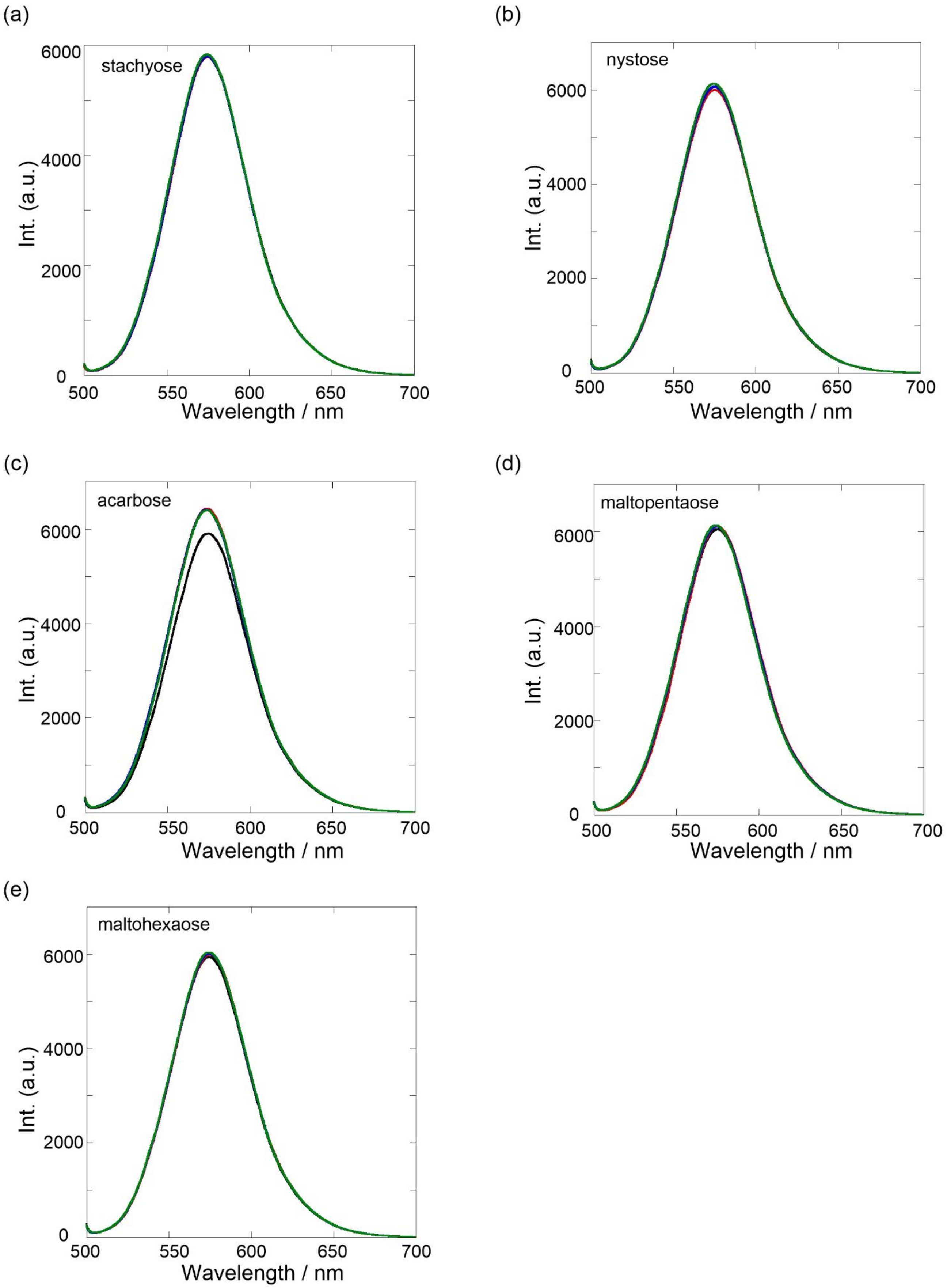

Figure S8. Fluorescence spectra (λ_{ex} 485 nm) of **Rhod-Cur$_{0.27}$** (78 μM in monomer units) in the absence (black) and presence of 100 μM stachyose (a), nystose (b), acarbose (c), maltopentaose (d), and maltohexaose (e) in 1:9 (v/v) DMSO-H_2O at room temperature; the fluorescence intensities were corrected by the absorbances at the excitation wavelength. Three measurements were performed for each sample (1st run; red, 2nd run; blue, and 3rd run; green).

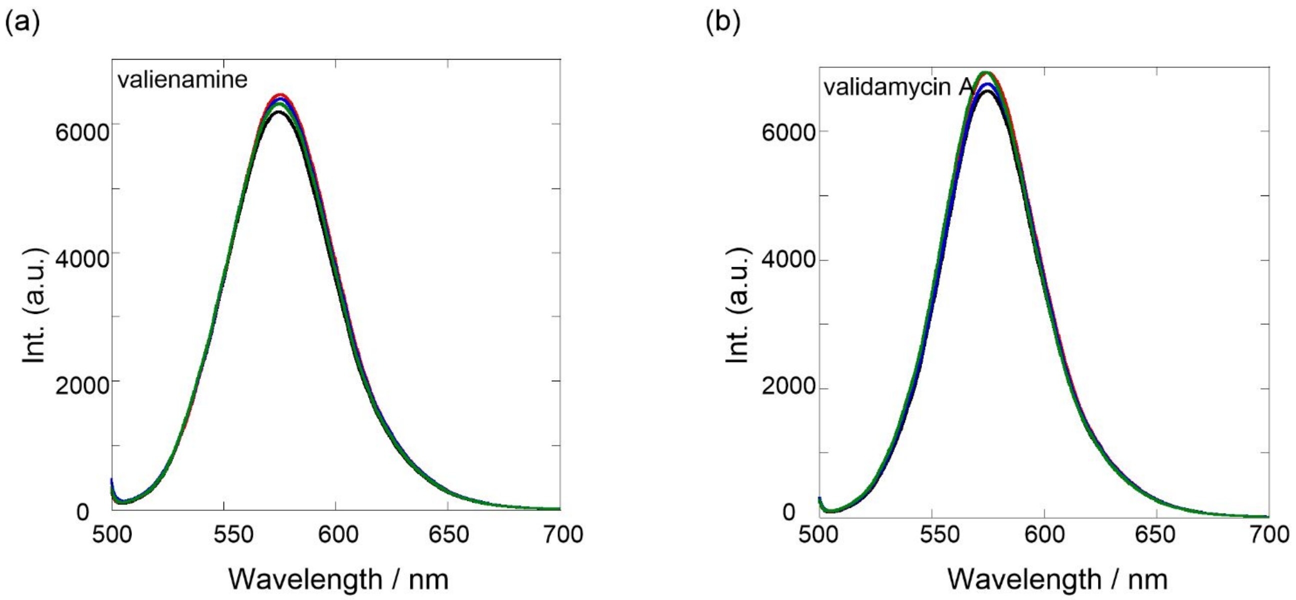

Figure S9. Fluorescence spectra (λ_{ex} 485 nm) of **Rhod-Cur$_{0.27}$** (80 μM in monomer units) in the absence (black) and presence of 100 μM valienamine (a) and validamycin A (b) in 1:9 (v/v) DMSO-H_2O at room temperature; the fluorescence intensities were corrected by the absorbances at the excitation wavelength. Three measurements were performed for each sample (1st run; red, 2nd run; blue, and 3rd run; green).

Titration of Glucosamine•HCl

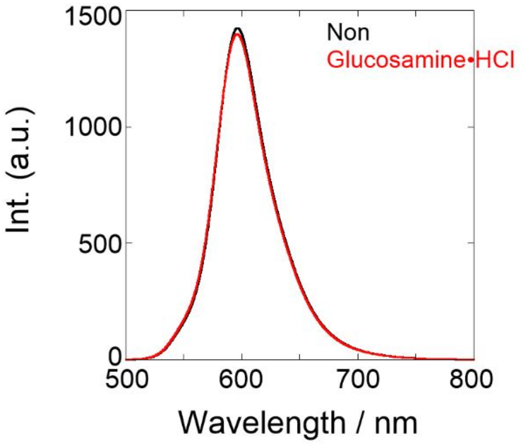

Figure S10. Fluorescence spectra (λ_{ex} 485 nm) of **Rhod-Cur$_{0.27}$** (87 μM in chromophore units) in the absence (black) and presence (red) of 112 μM glucosamine•HCl in 1:9 (v/v) DMSO-H_2O at room temperature; the excitation wavelength applied was those at which comparable absorbances were obtained.
